# State-dependent Gaussian kernel-based power spectrum modification for accurate instantaneous heart rate estimation

**DOI:** 10.1371/journal.pone.0215014

**Published:** 2019-04-05

**Authors:** Heewon Chung, Hooseok Lee, Jinseok Lee

**Affiliations:** Department of Biomedical Engineering, Wonkwang University College of Medicine, Iksan, Republic of Korea; Universitatsmedizin Greifswald, GERMANY

## Abstract

Accurate estimation of the instantaneous heart rate (HR) using a reflectance-type photoplethysmography (PPG) sensor is challenging because the dominant frequency observed in the PPG signal corrupted by motion artifacts (MAs) does not usually overlap the true HR, especially during high-intensity exercise. Recent studies have proposed various MA cancellation and HR estimation algorithms that use simultaneously measured acceleration signals as noise references for accurate HR estimation. These algorithms provide accurate results with a mean absolute error (MAE) of approximately 2 beats per minute (bpm). However, some of their results deviate significantly from the true HRs by more than 5 bpm. To overcome this problem, the present study modifies the power spectrum of the PPG signal by emphasizing the power of the frequency corresponding to the true HR. The modified power spectrum is obtained using a Gaussian kernel function and a previous estimate of the instantaneous HR. Because the modification is effective only when the previous estimate is accurate, a recently reported finite state machine framework is used for real-time validation of each instantaneous HR result. The power spectrum of the PPG signal is modified only when the previous estimate is validated. Finally, the proposed algorithm is verified by rigorous comparison of its results with those of existing algorithms using the ISPC dataset (*n* = 23). Compared to the method without MA cancellation, the proposed algorithm decreases the MAE value significantly from 6.73 bpm to 1.20 bpm (*p* < 0.001). Furthermore, the resultant MAE value is lower than that obtained by any other state-of-the-art method. Significant reduction (from 10.89 bpm to 2.14 bpm, *p* < 0.001) is also shown in a separate experiment with 24 subjects.

## Introduction

In recent years, instantaneous heart rate (HR) estimation has attracted considerable attention owing to the advent of wearable devices such as wristwatches and bands that can be used to obtain photoplethysmographs (PPGs). At present, various commercially available reflectance-type wrist-worn PPG devices, such as Apple Watch, Fitbit Surge, and Samsung Gear, are capable of producing instantaneous HR estimates. However, the accuracy of most of these devices is limited to situations in which the wearer is at rest, walking, or performing low-intensity exercise. During high-intensity exercise, the measured PPG signals are severely corrupted by motion artifacts (MAs) that are shaped similarly to pure pulses, which cause the dominant frequency in the PPG signal to deviate from the true HR. In addition, it is challenging to detect the pure pulse peak for estimating the HR. Here, severe corruption implies that motion artifacts with larger amplitude than the pure pulse are coupled with the PPG signal, making it difficult to distinguish the actual pure pulse [1–3].

Owing to this limitation, there is a need for wearable devices that produce accurate instantaneous HR estimates during high-intensity exercise. Such a need is further necessitated by the fact that, for instance, accurate real-time HR monitoring is required for efficient cardiac rehabilitation exercise, which requires the instantaneous HR to be maintained within the prescribed HR range [4, 5]. In addition, several studies have demonstrated the need for implementing effective HR dynamics during exercise for purposes such as glycemic control in individuals with type 1 diabetes [6], to not only avoid all-cause mortality and cardiac death [7–10] but also determine the capability of the autonomic system to respond to stressors [11–13].

Many algorithms have been proposed for achieving accurate HR estimation based on PPG signals [14–19], such as independent component analysis [14, 15], principle component analysis [16, 17], singular spectrum analysis [18, 19], and empirical mode decomposition [17] algorithms. These algorithms use signal processing techniques to separate uncorrelated signals from a set of mixed signals and yield accurate HR estimates under a wide range of conditions, including those of rest, walking, and light-intensity exercise. However, their performances have not been validated under high-intensity exercise. Furthermore, they adopt signal processing techniques without noise reference identification. Recently, many studies have focused on the use of simultaneously measured acceleration signals as noise references. This process facilitates efficient removal of MAs that corrupt the PPG signal, thereby allowing more accurate HR estimation during high-intensity exercise [1–3, 20–25]. Nevertheless, some results of these studies show significant deviation from the true HRs. For instance, in *subject 14* of the IEEE Signal Processing Cup (ISPC) 2015 dataset, the mean absolute errors (MAEs) were 9.59 bpm [3], 8.07 bpm [1], 7.29 bpm [21], and 6.63 bpm [20]. One reason for such deviation is that the employed acceleration signals do not always represent the true MAs. For example, when the subject taps his/her finger, twists his/her wrist, or closes/opens his/her fist, the measured PPG signal is distorted by the movement whereas the accelerometer may not capture the movement. In addition, high-intensity exercise may cause the MAs to overwhelm the PPG signals, resulting in a signal-to-noise ratio (SNR) that is too low for the identification of the dominant frequency corresponding to the true HR. Furthermore, when the dominant frequency determined from the acceleration signals overlaps the true HR, the HR information may be lost after MA cancellation. Thus, it is not always possible to achieve accurate HR estimation.

To address the above-mentioned issues, the present study proposes a method that significantly reduces inaccurate HR estimations through modification of the power spectrum of the PPG signals by emphasizing the power of the frequency corresponding to the true HR.

## Methods

### Ethics statement

This study was approved by the institutional review board of Wonkwang University, Republic of Korea (WKUIRB 201805-032-01). All the participants provided written informed consent.

### Dataset

In this study, we used two datasets, namely the ISPC dataset (*n* = 23) and the dataset obtained by our developed wearable device, i.e., the BAMI dataset (*n* = 24) [26]. Both datasets included multichannel PPG signals and multi-axis accelerometer signals simultaneously acquired by wrist-worn devices during physical exercise. In addition, chest ECG signals were simultaneously recorded using wet ECG sensors. The ECG-based HRs were considered to be true values; they were calculated using 8-s windows with 2-s shifts (6-s overlap), yielding HRs every 2 s. The same window length (8 s) and shift (2 s) were used throughout this study to assess the performance of the proposed algorithm against that of existing algorithms [1–3, 20–25, 27].

The ISPC dataset comprises two-channel PPG signals and three-axis acceleration signals sampled at 125 Hz, and it is publicly downloadable [28]. The data correspond to three exercise types: Type 1 (T1), Type 2 (T2), and Type 3 (T3). The T1 dataset (*n* = 12) includes the data of each subject running on a treadmill at varying speeds: 30 s of rest, 1 min at 6–8 km/h, 1 min at 12–15 km/h, 1 min at 6–8 km/h, 1 min at 12–15 km/h, and another 30 s of rest. The T2 dataset (*n* = 5) includes the data of each subject performing a variety of actions, including running, jumping, push-ups, shaking hands, stretching, and pushing. The T3 dataset (*n* = 6) includes the data of each subject performing high-intensity arm movements, such as boxing. The true HR was calculated from the ECG signals by manually identifying the individual R peaks in each time window. No R-peak detection algorithm was used to avoid any possible detection error.

**Fig 1:**
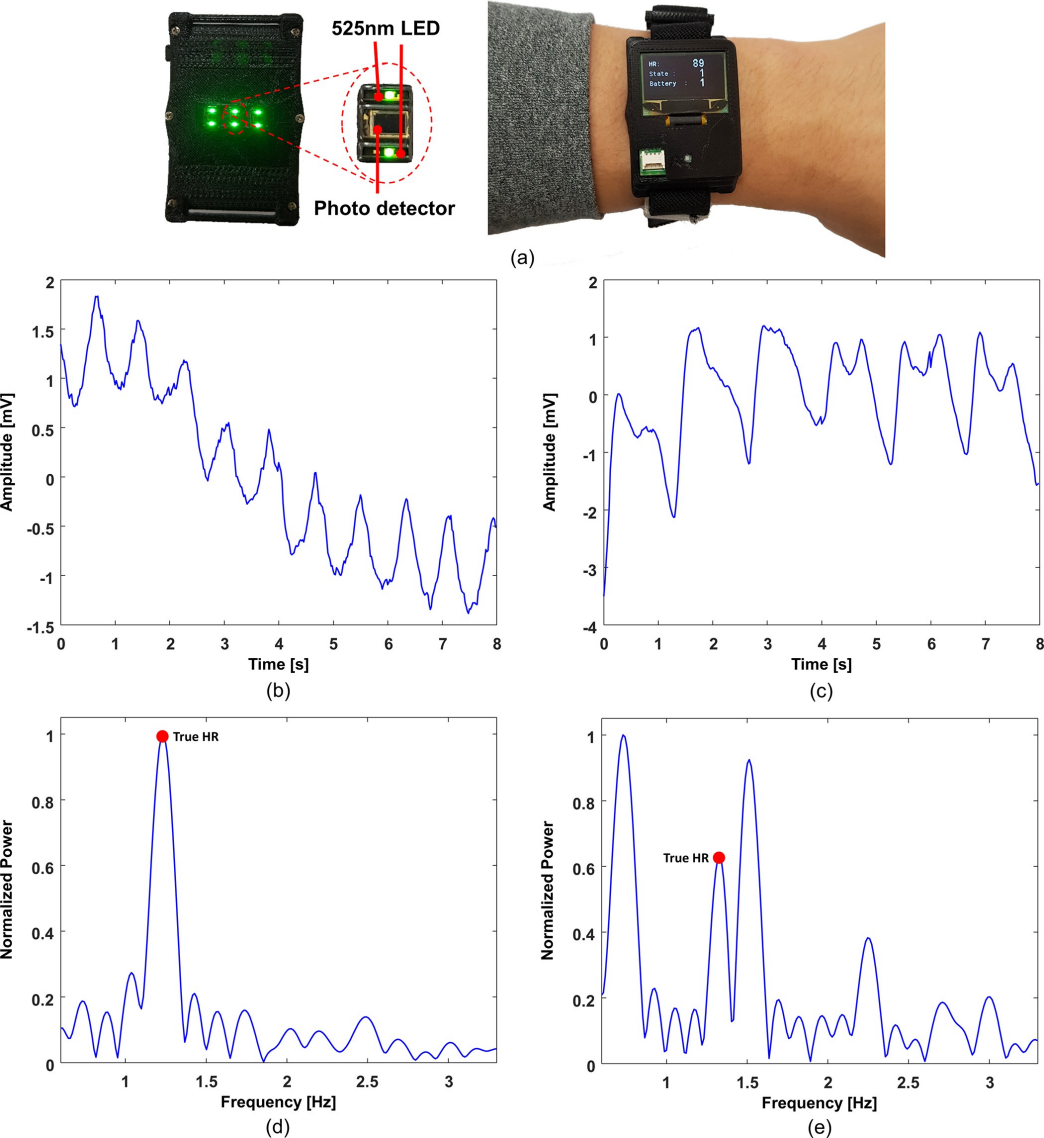
Wearable BAMI device for acquiring three-channel PPG signals and three-axis acceleration signals. (a) device, (b) example of measured PPG signal corrupted by low-intensity MAs, (c) example of measured PPG signal corrupted by high-intensity MAs, (d) power spectrum of signal (b) with true HR, and (e) power spectrum of signal (c) with true HR.

The BAMI dataset comprises three-channel PPG signals and three-axis acceleration signals sampled at 50 Hz. Fig 1(A) shows the BAMI device, which consists of three photosensors (NJL5310R, NJR Corporation, Japan) for acquiring the PPG signals and an inertial measurement unit (IMU; LSM6DSMUSTR, STMicroelectronics) for acquiring the acceleration signals. The BAMI dataset is also publicly downloadable [26]. Fig 1(B) and 1(C) show examples of the measured PPG signals corrupted by low- and high-intensity MAs, respectively. Fig 1(D) and 1(E) show the corresponding power spectra. It can be seen that the dominant frequency in the power spectrum corresponds to the true HR in the case of low-intensity MAs. On the other hand, the dominant frequency may not correspond to the true HR in the case of high-intensity MAs. The data were collected from 24 healthy subjects (10 male, 14 female; average age: 26.9±4.8 years) at Wonkwang University, recruited by trained personnel. The exercise protocol included 1 min of rest, 2 min of walking for warming up, 3 min of running at 6–8 km/h, 2 min of walking, 3 min of running at 8–12 km/h, and 1 min of walking for cooling down. The entire process was executed on a treadmill. For the reference true HRs, ECG data were simultaneously recorded at a sampling rate of 125 Hz by a 24-h Holter monitor (SEER Light, GE Healthcare, Milwaukee, WI, USA). Then, we manually identified the R peaks and computed the average RR intervals in each time window. Note that in the ISPC dataset, the number of cardiac cycles was manually counted in each 8-s window with 2-s shifts from the measured ECG signals [28].

### Preprocessing

In this study, the instantaneous HR *HR*_*est*_(*i*) was estimated on the basis of the 8-s segmented PPG signals ***S***_*np*_(*i*) and the acceleration signals ***A***_*m*_(*i*) in the *i*^th^ window, where *np* = {1, 2,… *NP*} (*NP* is the number of photosensors) and *m* = {1, 2,… *M*} (*M* is the number of acceleration axes). HR estimation was performed every 2 s (2-s shift; thus, 6-s overlap). To assess the performance of our algorithm, we used the same window length and shift as those used in previous studies [1–3, 20–25, 27]. A fourth-order Butterworth bandpass filter (BPF) with cutoff frequencies of 0.4 and 4 Hz was applied to the signals ***S***_*np*_(*i*). The range of approximately 40–200 bpm covers the HRs of subjects of all ages, both at rest and during high-intensity physical activity [29, 30]. The filtered signals were subsequently normalized to zero mean with unit variance in the *i*^th^ window. The normalized signals were then averaged and down-sampled to 25 Hz to reduce the computational load. The power spectrum was subsequently computed by 2,048-point fast Fourier transformation (FFT) to provide high-frequency resolution (12.5 Hz/2,048 = 0.0061 Hz; 0.3662 bpm), and the results were normalized to have a minimum value of zero and maximum value of one, denoted by ***P***_*S*_(*i*). Given a signal with a sampling rate of 125 Hz (without down-sampling), 8,192-point FFT is required to provide a similar frequency resolution (62.5 Hz/8,192 = 0.0076 Hz). The same steps (BPF, down-sampling, and normalization) were applied to the signals ***A***_*m*_(*i*), and their normalized 2,048-point FFTs were denoted by ***P***_*A*_(*i*).

### HR estimation with MA cancellation

Given the computed power spectra ***P***_*S*_(*i*) and ***P***_*A*_(*i*), the true clean power spectrum ***P***_*C*_(*i*) can be estimated as

$$P_{C}(i)\approx P_{S}(i)-P_{A}(i)$$ (1)

Eq (1) can also be expressed as

$$P_{C}(i)\approx P_{S}(i)-P_{A}(i)=(\frac{P_{S}(i)-P_{A}(i)}{P_{S}(i)})P_{S}(i)$$ (2)

By substituting ***P***_*S*_(*i*) in the term $\left(\frac{P_{S}(i)-P_{A}(i)}{P_{S}(i)}\right)$ in Eq (2) with ***P***_*C*_(*i*)+***P***_*A*_(*i*), we have

$$P_{C}(i)\approx \left(\frac{P_{C}(i)}{P_{C}(i)+P_{A}(i)}\right)P_{S}(i)$$ (3)

Assuming that the power spectra ***P***_*C*_(*i*−1) and ***P***_*C*_(*i*) in consecutive windows nearly overlap, ***P***_*C*_(*i*) in the term $\left(\frac{P_{A}(i)}{P_{C}(i)+P_{A}(i)}\right)$ in Eq (3) can be substituted with ***P***_*C*_(*i*−1) obtained in the (*i*-1)^th^ window. Hence, ***P***_*C*_(*i*) can be recursively estimated as

$$P_{C}(i)=\left(\frac{P_{C}(i-1)}{P_{C}(i-1)+P_{A}(i)}\right)P_{S}(i)$$ (4)

Finally, we determined the dominant frequency in ***P***_*C*_(*i*) for HR values of 0.6–3.3 Hz (approximately 40–200 bpm) to obtain *HR*_*est*_(*i*). It is shown that the power spectrum ***P***_*C*_(*i*) is recursively obtained from the previous power spectrum ***P***_*C*_(*i*−1) in the (*i-1*)^th^ window. It is important to consider the previous power spectrum in order to efficiently suppress MAs, because MAs originate from dynamic frequency changes with higher uncertainties whereas clean PPG signals change slowly, assuming that the HRs in consecutive windows are close. It can be shown that the recursive estimation is similar to the Bayesian approach in that it estimates the current HR density function on the basis of the previous density function. On the other hand, it differs from the Bayesian approach in that its prediction is not based on the HR transition model.

**Fig 2:**
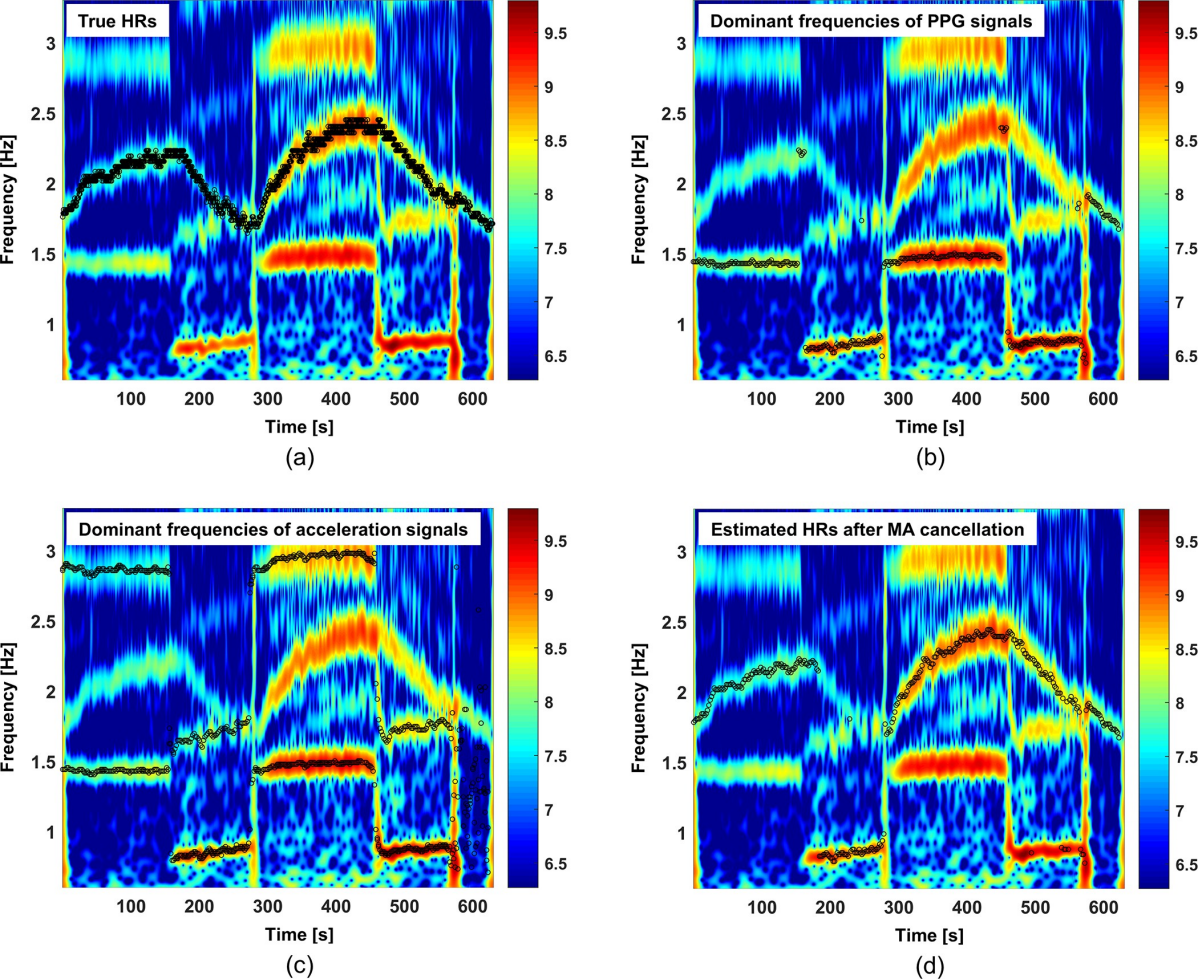
Time-frequency spectrum (TFS) of the PPG signals for *subject 1* obtained by the BAMI device. (a) true HRs (black circles) on the TFS, (b) dominant frequencies of the PPG signals (black circles) on the TFS, (c) dominant frequencies of the three-axis acceleration signals (black circles) on the TFS, and (d) dominant frequencies of the PPG signals (black circles) on the TFS after MA cancellation using the acceleration signals.

Fig 2 shows time-frequency spectrum (TFS) of the PPG signals for *subject 1* measured by the BAMI device. In Fig 2(A), the black circles represent the true HRs, and it can be seen that some frequencies from the measured PPG signals reflect the true HRs. In Fig 2(B), the black circles represent the dominant frequencies of the PPG signals, and it can be seen that they do not overlap the true HRs. Hence, if the HR is estimated by directly identifying the dominant frequency in the power spectrum ***P***_*S*_(*i*), the result would be inaccurate. In Fig 2(C), the black circles represent the dominant frequencies of the three-axis acceleration signals, and they indicate the detection of MAs by the power spectrum ***P***_*A*_(*i*). In Fig 2(D), the black circles represent the estimated HRs after MA cancellation using the acceleration signals. The results show that the use of ***P***_*C*_(*i*) after MA cancellation improves the accuracy of HR estimation.

For more rigorous comparison and analysis, the performances of the proposed algorithm with and without MA cancellation were compared for both the ISPC dataset and the BAMI dataset. The results are summarized in Table 1A and 1B. It can be seen that the use of ***P***_*C*_(*i*) significantly decreases the MAE from 13.71 to 6.73 bpm and from 21.27 to 10.89 bpm for the ISPC dataset and the BAMI data, respectively. The overall MAE from both datasets decreases from 17.57 bpm to 8.86 bpm, which is summarized in Table 2. However, MA cancellation has an inherent limitation and does not always work for all data. It is inapplicable under certain conditions, such as when the MAs are uncorrelated with the acceleration signals, the SNR is extremely low, and the overlap between the true HR and the MA frequency is extremely small. For instance, in the cases of *subjects 10*, *14*, *15*, *17*, and *20* in the ISPC dataset and *subjects 5*, *6*, *9*, *11*, *16*, and *18* in the BAMI dataset, the MAEs were extremely high both with and without MA cancellation. These issues were also observed in other previously reported algorithms [1–3, 20–25].

**Table 1:** Comparison of the HR estimation results obtained with and without MA cancellation for the ISPC (*n* = 23) and BAMI (*n* = 24) datasets. The performance was evaluated on the basis of the mean absolute error (MAE).

A				B			
Dataset	Subject	Without MA cancellation	With MA cancellation	Dataset	Subject	Without MA cancellation	With MA cancellation
ISPC	1	8.28	3.81	BAMI	1	46.64	11.86
	2	24.44	2.88		2	29.46	9.00
	3	16.77	1.15		3	17.61	4.86
	4	7.27	1.09		4	36.59	17.31
	5	2.09	0.79		5	39.88	25.49
	6	4.67	1.48		6	39.29	33.06
	7	1.36	4.09		7	6.83	3.90
	8	3.79	0.65		8	9.75	4.81
	9	0.43	0.43		9	20.42	21.30
	10	35.70	21.60		10	33.71	10.56
	11	10.66	2.73		11	34.01	31.28
	12	13.50	1.22		12	17.76	6.58
	13	15.68	8.70		13	13.13	3.15
	14	19.07	20.47		14	5.94	3.62
	15	10.06	15.27		15	13.51	3.30
	16	12.86	7.40		16	18.65	14.71
	17	27.00	15.48		17	27.29	8.59
	18	15.40	2.31		18	27.49	15.55
	19	24.91	9.39		19	16.28	3.95
	20	24.95	21.21		20	15.72	3.17
	21	22.27	8.87		21	4.77	4.17
	22	13.50	3.12		22	8.70	2.42
	23	0.70	0.70		23	19.67	12.54
					24	7.44	6.17
**Average**		**13.71**	**6.73**	**Average**		**21.27**	**10.89**

**Table 2:** Comparison summary of the HR estimation results obtained with and without MA cancellation. Each result was obtained from all datasets (*n* = 47): ISPC (*n* = 23) and BAMI (*n* = 24) datasets. The performance was evaluated on the basis of the mean absolute error (MAE).

**All (Nos. 1–47)**	**Without MA cancellation**	**With MA cancellation**
	**17.57**	**8.86**

### Result validation using FSM framework

**Fig 3:**
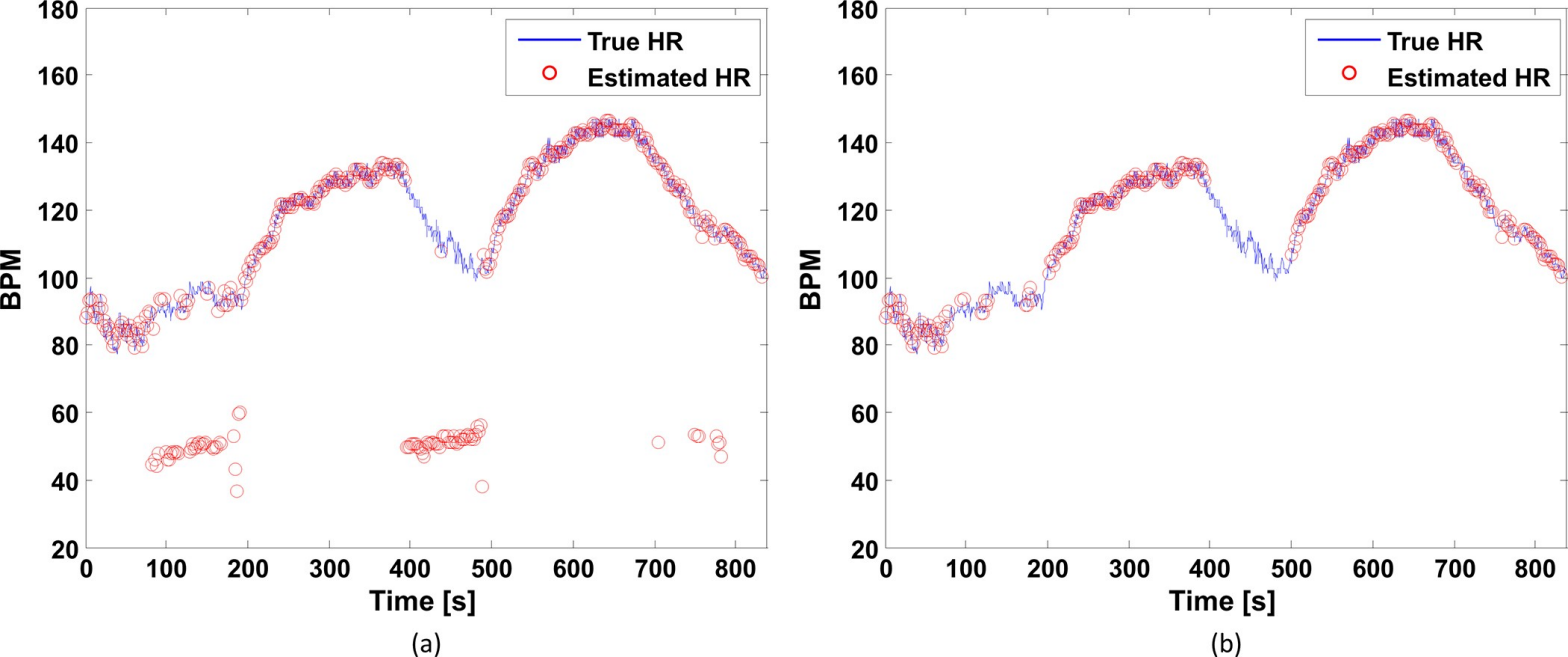
Comparison of HR estimation results. (a) with and (b) without the FSM framework for *subject 1* in the BAMI dataset.

The FSM framework was used to eliminate inaccurate estimation results and thus overcome the above-mentioned limitation. Four states are defined in the framework, namely stable, recovery, alert, and uncertain states. After the estimation of *HR*_*est*_(*i*) with MA cancellation, the FSM framework is used to determine the state and validate the estimation result in real time. A stable state indicates that the estimated HR is highly likely to be accurate and it is thus declared valid. A recovery state indicates that the estimated HR is somewhat likely to be accurate with the need to explore possible transition to the stable state. An alert state indicates that the estimated HR is somewhat likely to be inaccurate. An uncertain state indicates that the estimated HR is highly likely to be inaccurate. The FSM framework transits from one state to another in every window in response to the estimation accuracy indicators, namely the crest factor (CF) and the HR change between consecutive windows. The CF is the ratio of the dominant frequency power to the root mean square of the total power of ***P***_*S*_(*i*). The higher the CF, the less corrupted is the PPG signal. Thus, the CF condition *CF*(*i*)≥*TH*_*CF*_ indicates that ***P***_*S*_(*i*) is acceptable for HR estimation, where *CF*(*i*) is the CF value in the *i*^th^ window and *TH*_*CF*_ is the CF threshold value. The HR change between consecutive windows is based on the observation that the absolute HR difference every 2 s is approximately 5 bpm at the 99% level in the ISPC database. Thus, the HR change condition |*HR*_*est*_(*i*)−*HR*_*est*_(*i*−1)|≤*TH*_*HR*_, where *TH*_*HR*_ is the HR change threshold, indicates acceptable estimation of *HR*_*est*_(*i*) on the basis of *HR*_*est*_(*i*−1) in the *(i-1)*^th^ window. The FSM framework thus determines the state on the basis of the CF and HR change, and only a stable state result is declared valid. Other state results are declared invalid and discarded. Accordingly, the FSM automatically validates the estimation results without the true HR value and ignores inaccurate estimation results caused by extremely low SNRs in the PPG signals or by MAs uncorrelated with the accelerometer signals. The details of the framework are presented elsewhere [27]. Fig 3 compares the results obtained with and without the FSM framework for *subject 1* in the BAMI dataset. As can be observed from Fig 3(A), MA cancellation does not always produce accurate HR estimates, while Fig 3(B) shows that the FSM framework successfully identifies valid HR estimates.

### Gaussian kernel-based power spectrum modification

**Fig 4:**
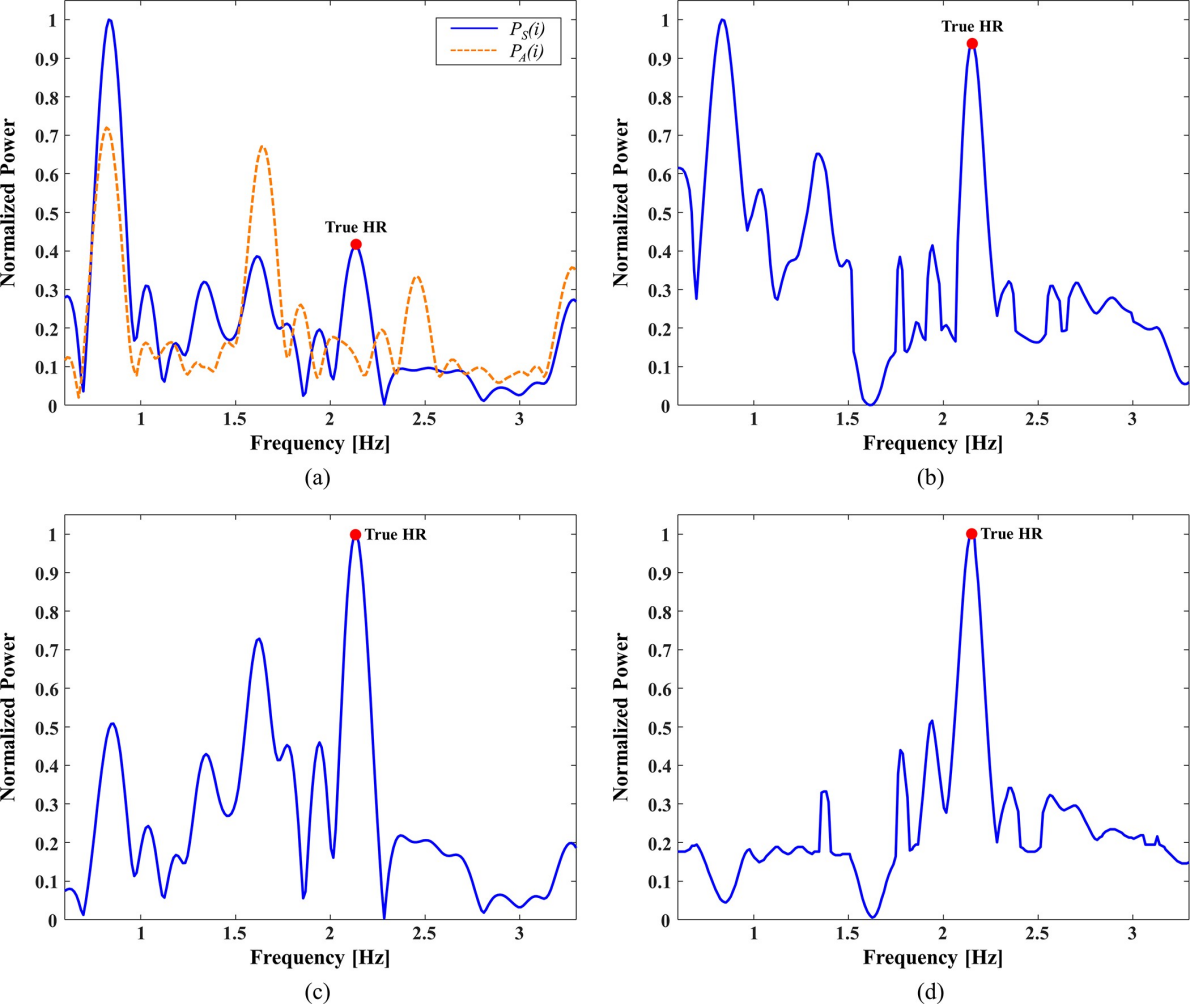
Sample HR estimation results obtained with MA cancellation. (a) power spectrum *P*_*S*_(*i*) (blue solid line) and power spectrum *P*_*A*_(*i*) (yellow dotted line), (b) power spectrum *P*_*C*_(*i*) after MA cancellation, (c) Gaussian kernel-based modified power spectrum $\hat{P_{S}}(i)$, and (d) power spectrum $\hat{P_{C}}(i)$ after MA cancellation based on $\hat{P_{S}}(i)$.

Fig 4 shows sample HR estimation results without (a) and with (b) MA cancellation for a certain window with a true HR of 2.1 Hz. Fig 4(A) shows the power spectrum ***P***_*S*_(*i*) obtained from the measured PPG signals before MA cancellation. The highest frequency peak occurs at 0.8 Hz, followed by 2.1 Hz (the true HR), 1.6 Hz, and 1.4 Hz. Fig 4(B) shows the power spectrum ***P***_*A*_(*i*) obtained from the simultaneously measured acceleration signals. The power spectrum ***P***_*A*_(*i*) has two dominant frequencies of 0.8 and 1.6 Hz, which overlap the MA frequencies in the power spectrum ***P***_*S*_(*i*), indicating that the acceleration signals correctly reflect the MA frequencies in the PPG signals. However, even after MA cancellation, the most dominant frequency in the power spectrum ***P***_*C*_(*i*) is 0.8 Hz, as shown in Fig 4(C), whereas the frequency power at the true HR (2.1 Hz) appears as a slightly weaker peak. Even if the MA frequencies were correctly identified using the acceleration signals and the corresponding MAs were removed, the MA frequency power was still greater than any other frequency power. This is because the MA frequency power in the power spectrum ***P***_*S*_(*i*) overwhelmed the frequency power of the true HR; thus, the MA frequency power was slightly greater than the frequency power of the true HR in the power spectrum ***P***_*C*_(*i*). Such inaccurate estimates are often observed when the SNR is extremely low, with the pure PPG signals overwhelmed by the MAs. When the FSM framework is applied under these conditions, the estimate may be declared invalid and ignored, assuming that *HR*_*est*_(*i*−1) is accurate. This would prevent degradation of the estimation accuracy, although the valid HR rate (VHR) would decrease.

To overcome the low SNR problem, the power spectrum ***P***_*S*_(*i*) was modified by emphasizing the power of the frequency corresponding to *HR*_*est*_(*i*−1), assuming that it is accurate. As expressed by Eq (5), a modified power spectrum denoted by $\hat{P_{S}}(i)$ was then obtained by multiplying the power spectrum ***P***_*S*_(*i*) with the Gaussian kernel function, which has a mean value of *HR*_*est*_(*i*−1).

$$\hat{P_{S}}(i)≔P_{S}(i)∙{\left(2\pi \right)}^{-\frac{1}{2}}∙exp\left(-\frac{{\left({HR}_{est}(i-1)\right)}^{2}}{2\sigma^{2}}\right)$$ (5)

where *HR*_*est*_(*i*−1) is assumed to be accurate. Otherwise, $\hat{P_{S}}(i)=P_{S}(i)$. The standard deviation was set to *σ* = 1 Hz or 60 bpm and its effect was investigated, as discussed in the “Results” section below. Fig 4(D) shows the Gaussian kernel-based modified power spectrum $\hat{P_{S}}(i)$. By emphasizing the power of the frequency corresponding to *HR*_*est*_(*i*−1), the highest peak of the power spectrum $\hat{P_{S}}(i)$ occurs at the true HR frequency of 2.1 Hz. Furthermore, after MA cancellation, the resultant clean power spectrum $\hat{P_{C}}(i)$ has a much more dominant peak at the true HR frequency, as shown in Fig 4(E). The FSM framework validates the HR estimate in real time and the Gaussian kernel-based modification is only applied when *HR*_*est*_(*i*−1) is declared valid. In addition, the modification requires the assumption that the HRs in two consecutive windows are close. This assumption was satisfied in the present study because the HR estimation was performed within 8-s windows with 2-s shifts (6-s overlap).

**Fig 5:**
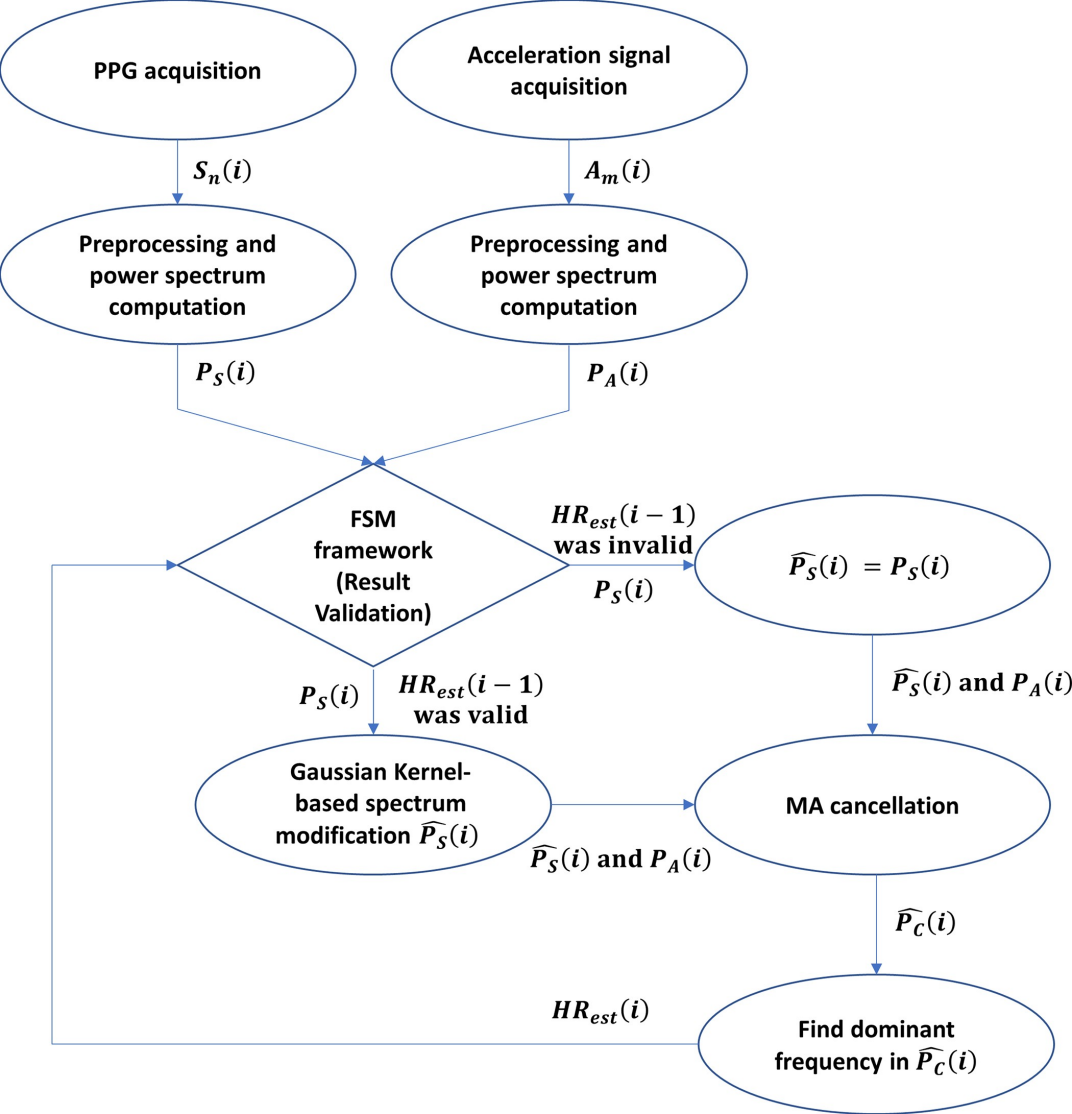
Flowchart of the proposed algorithm.

The flowchart of the proposed algorithm is shown in Fig 5. In the *i*^th^ window, the PPG and acceleration signals (i.e., ***S***_*n*_(*i*) and ***A***_*m*_(*i*)) are acquired, and the power spectra (***P***_*S*_(*i*) and ***P***_*A*_(*i*)) are computed in the preprocessing stage. If the FSM framework declares *HR*_*est*_(*i*−1) to be valid in the (*i-*1)^th^ window, ***P***_*S*_(*i*) is modified using the Gaussian kernel function in Eq (5). Otherwise, $\hat{P_{S}}(i)=P_{S}(i)$. The clean power spectrum $\hat{P_{C}}(i)$ is subsequently computed with MA cancellation based on $\hat{P_{S}}(i)$ and ***P***_*A*_(*i*). Finally, *HR*_*est*_(*i*) is obtained by identification of the dominant frequency in $\hat{P_{C}}(i)$ over the HR range 0.6–3.3 Hz and validated by the FSM framework.

### Evaluation metrics

The performance of the proposed algorithm was evaluated by comparing its results with those of previously developed algorithms [1–3, 20–25, 27] using the ISPC (*n* = 23) and BAMI (*n* = 24) datasets. It should be noted that *N* = 2 for the ISPC dataset and *N* = 3 for the BAMI dataset. For both datasets, *M* = 3. Two methods were adopted to evaluate the HR estimation performance of the proposed algorithm using state-dependent Gaussian kernel-based power spectrum modification with the FSM framework (FSM-SGPS). The first method involved direct frequency determination of the dominant frequency (DFDF) in the power spectrum ***P***_*C*_(*i*) after MA cancellation. The second method involved a combination of the FSM framework and DFDF (FSM-DFDF). The accuracy of the algorithm was further evaluated by calculating the absolute error (AE) of its estimation:

$$AE(i)=|{HR}_{est}(i)-{HR}_{true}(i)|,$$ (6)

where *HR*_*true*_(*i*) is the true HR (bpm) in the *i*^th^ window. The overall evaluation of HR estimation was performed on the basis of the MAE (bpm), average of the relative AEs (ARE) (%), and valid HR rate (VHR) (%) as the percentage of valid results among all windows:

$$MAE=\frac{\sum_{i=1}^{N_{valid}}AE(i)}{N_{valid}}$$ (7)

$$ARE=\frac{\sum_{i=1}^{N_{valid}}\frac{AE(i)}{{HR}_{true}(i)}\times 100}{N_{valid}}$$ (8)

$$VHR=\frac{N_{valid}\times 100}{N_{window}}$$ (9)

where *N*_*window*_ is the total number of windows used for HR estimation and *N*_*valid*_ is the number of windows declared valid by the FSM framework. In accordance with the previous FSM framework-based study [27], we used *TH*_*CF*_ = 2.4 and *TH*_*HR*_ = 5.03 bpm. In addition, for statistical analysis, we performed one-way analysis of variance (ANOVA) using MATLAB (MathWorks, Natick, MA, USA) to compare the resultant HR means from different algorithms in order to obtain statistical evidence as to whether the associated means are significantly different. A *p* value below 0.05 was considered significant.

### Optimal parameter search

**Fig 6:**
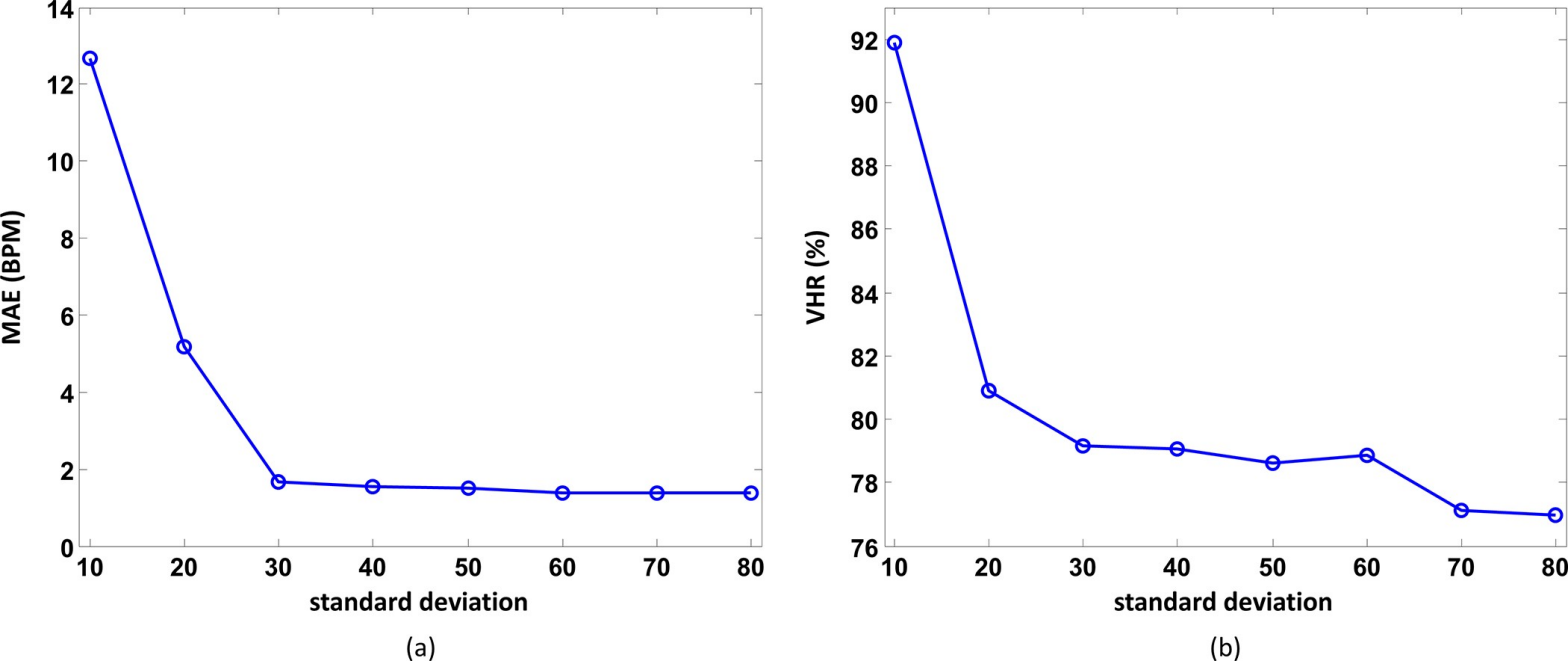
Variation. (a) MAE and (b) VHR with *σ* in the Gaussian function given by Eq (5).

To determine the optimal value of the standard deviation *σ* in Eq (5), various values between 10 bpm and 80 bpm in increments of 10 bpm were used to apply the proposed FSM-SGPS algorithm to all the 23 subjects of the ISPC dataset. Fig 6(A) and 6(B) show the estimation MAEs and VHRs with respect to *σ*. It can be seen that the MAE drastically decreases as *σ* increases from 10 to 30 bpm and then becomes constant as *σ* further increases to 80 bpm. Similarly, VHR drastically decreases as *σ* increases from 10 to 30 bpm and then becomes constant as *σ* further increases to 60 bpm. The optimal value of *σ* may vary with the subject, exercise type, and exercise intensity, but the performance is nearly the same for *σ* values of 30–60 bpm. Hence, *σ* = 60 bpm was adopted for further performance analysis.

## Results

### Results for ISPC data

**Fig 7:**
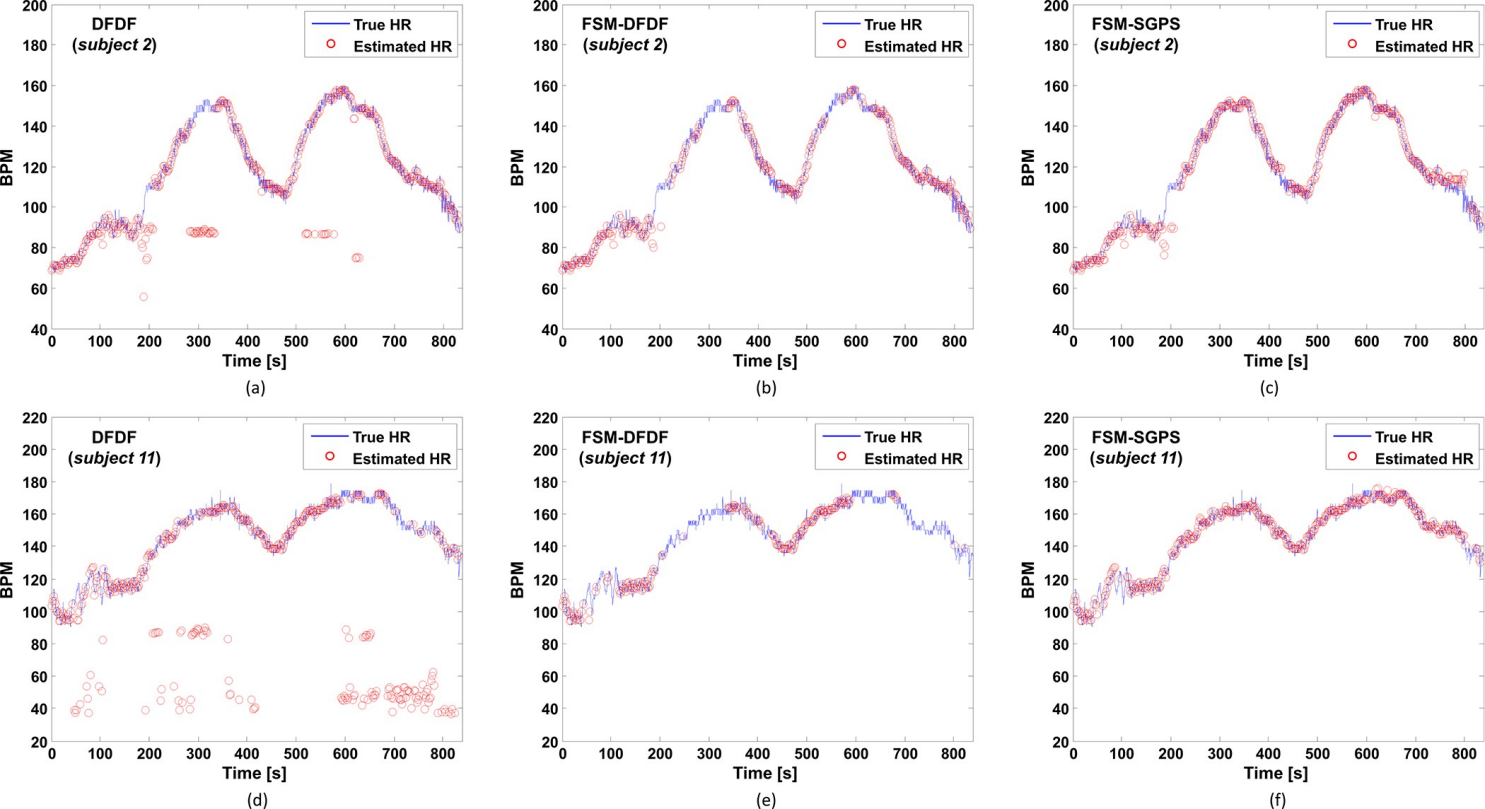
Estimated HR trace comparison for two subjects of the ISPC dataset, obtained by DFDF (with MA cancellation only), FSM-DFDF (with MA cancellation and FSM framework), and FSM-SGPS (the proposed algorithm). (a) DFDF results of *subject 2*, (b) FSM-DFDF results of *subject 2*, (c) FSM-SGPS results of *subject 2*, (d) DFDF results of *subject 11*, (e) FSM-DFDF results of *subject 11*, (f) FSM-SGPS results of *subject 11*.

Fig 7 shows the HR trace comparison for the three HR estimation methods, namely DFDF, FSM-DFDF, and FSM-SGPS, applied to the PPG data of *subjects 2* and *11* of the ISPC dataset. It can be seen that FSM-SGPS produces more accurate estimates compared with DFDF and higher VHR results compared with FSM-DFDF.

Table 3 summarizes the overall performance comparison of the three estimation methods. Applying the FSM-SGPS method to all the subjects of the ISPC dataset yielded an MAE of 1.20 bpm, an ARE of 1.05%, and a VHR of 78.84%. Thus, the MAE was 0.21 bpm higher than that of FSM-DFDF but 82.17% lower (-5.51 bpm) than that of DFDF. The FSM-SGPS results were statistically different from the DFDF results (*p* < 0.001) but not significantly different from the FSM-DFDF results. However, the VHR of the proposed algorithm was much higher (21.40% higher) than that of FSM-DFDF. This implies that FSM-SGPS minimizes the invalidity rate of the HR results by increasing the overall accuracy even under severe MA corruption, while FSM-DFDF ignores the results as far as possible on the basis of the CF and the HR condition. For instance, for *subjects 10*, *13*, *14*, *15*, *17*, *18*, *19*, *20*, and *21*, the VHRs of FSM-DFDF were less than 50% (average = 26.9%), indicating that more than 50% of the results were ignored. For the same subjects, FSM-SGPS increased the average VHR to 60.03% by minimizing the invalidity rate of the HR results.

**Table 3:** Performance comparison of the DFDF, FSM-DFDF, and FSM-SGPS HR estimation methods.

Subject	DFDF		FSM-DFDF			FSM-SGPS		
	MAE (bpm)	ARE (%)	MAE (bpm)	ARE (%)	VHR (%)	MAE (bpm)	ARE (%)	VHR (%)
**1**	3.81	2.89	0.80	0.68	62.16	0.80	0.68	77.03
**2**	2.88	2.54	0.79	0.66	59.46	0.94	0.87	83.11
**3**	1.15	0.87	0.54	0.41	82.14	0.61	0.49	96.43
**4**	1.09	1.05	0.72	0.58	81.51	0.81	0.68	82.88
**5**	0.79	0.62	0.59	0.43	86.30	0.78	0.56	96.58
**6**	1.48	1.25	0.87	0.69	73.33	1.39	1.08	96.67
**7**	4.09	2.72	0.66	0.52	73.43	0.77	0.58	93.01
**8**	0.65	0.53	0.63	0.52	87.50	0.62	0.51	96.88
**9**	0.43	0.35	0.43	0.35	96.64	0.44	0.36	100.00
**10**	21.60	13.10	1.44	0.98	18.79	1.70	1.06	57.05
**11**	2.73	1.90	1.12	0.72	78.32	1.17	0.76	96.50
**12**	1.22	0.85	0.96	0.68	64.38	0.96	0.68	93.15
**13**	8.70	9.48	2.51	2.85	28.97	2.86	3.28	77.57
**14**	20.47	26.94	0.60	0.99	5.63	3.93	5.78	14.08
**15**	15.27	18.87	0.83	1.21	36.50	1.32	1.69	74.45
**16**	7.40	6.75	1.04	0.82	63.89	1.40	1.19	71.53
**17**	15.48	9.65	1.67	1.26	17.76	2.87	2.04	46.05
**18**	2.31	1.92	1.07	0.87	34.65	1.91	1.61	60.40
**19**	9.39	6.83	0.89	0.67	36.94	1.09	0.81	78.98
**20**	21.21	22.45	1.35	1.64	30.30	1.49	1.65	70.45
**21**	8.87	6.14	1.23	0.89	33.10	1.98	1.47	61.27
**22**	3.12	2.53	1.35	1.07	69.42	1.32	1.04	89.26
**23**	0.70	0.82	0.70	0.82	100.00	0.70	0.82	100.00
**T1**	**3.49**	**2.39**	**0.80**	**0.60**	**71.99**	**0.89**	**0.67**	**89.11**
**T2**	**13.27**	**15.71**	**1.20**	**1.50**	**40.28**	**1.64**	**1.96**	**67.31**
**T3**	**7.76**	**5.64**	**1.20**	**0.93**	**42.63**	**1.65**	**1.28**	**67.92**
**All**	**6.73**	**6.13**	**0.99**	**0.88**	**57.44**	**1.20**	**1.05**	**78.84**

Table 4 summarizes the performance comparison of the proposed method and other previously proposed methods. It should be noted that some of the previous methods were tested for only the first 12 subjects of the ISPC dataset, while the others considered *subjects 14–23*. Only a few methods considered all 23 subjects. For a more accurate comparison, the test results are shown for subgroups of the study participants (*subjects 1–12*, *subjects 13–23*, *subjects 14–23*, all subjects except *subject 13*, and all 23 subjects). The results indicate that the FSM-SGPS algorithm yields more accurate HR estimates than all the other methods.

**Table 4:** Comparison of MAEs of various HR estimation methods for the ISPC dataset.

Subject	TROIKA [20]	JOSS [1]	EEMD [2]	SpaMa [21]	IMAT [22]	Spectrap [23]	WFPV [3]	SVD [24]	PF [25]	FSM-SGPS MAE (VHR)
1–12	2.34	1.28	1.02	0.89	1.25	1.50	1.02	0.94	1.17	**0.89 (89.11)**
13–23	-	-	-	3.36	-	-	3.01	-	-	**1.64 (67.64)**
14–23	3.19	3.05	-	3.35	-	2.13	2.95	-	-	**1.50 (66.65)**
All except subject 13	2.73	2.08	-	2.01	-	1.79	1.90	-	-	**1.13 (78.90)**
All 1–23	-	-	-	2.07	-	-	1.97	-	-	**1.20 (78.84)**

**Fig 8:**
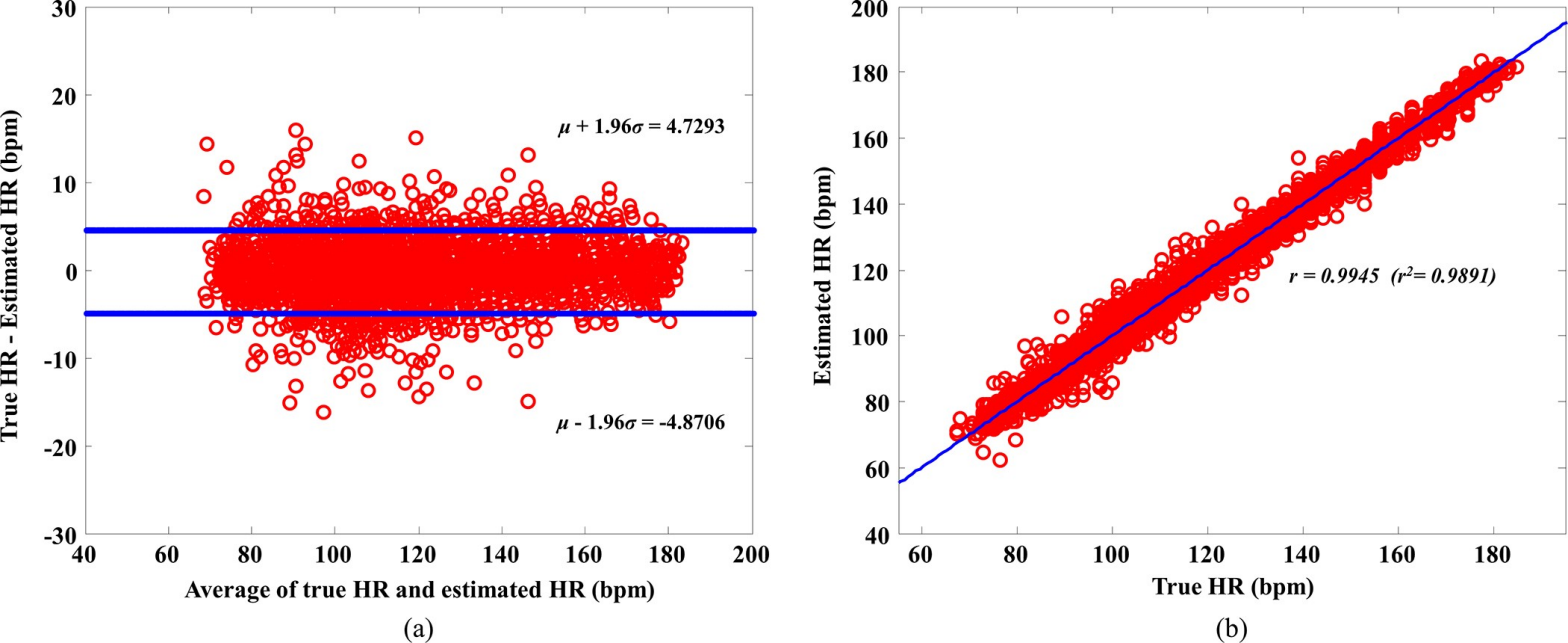
Bland–Altman plot and correlation. (a) Bland–Altman plot of estimated and true HRs and (b) correlation for the proposed FSM-SGPS algorithm.

Fig 8(A) shows a Bland–Altman plot, from which the limit of agreement can be seen to lie between –4.87 bpm and 4.72 bpm (mean of -0.10 bpm and standard deviation of 2.22 bpm). Fig 8(B) shows the scatter plot of the valid HRs estimated by the FSM-SGPS algorithm and the true HRs. The Pearson correlation coefficient was determined to be 0.9945 (*r*^2^ = 0.9891).

### Results for BAMI dataset

Table 5 summarizes the HR estimation performance comparison of the three above-mentioned methods for the BAMI dataset. For the 24 subjects, FSM-SGPS yielded an MAE of 2.14 bpm, an ARE of 1.73%, and a VHR of 90.48%; DFDF yielded an MAE of 10.89 bpm and an ARE of 8.32%; and FSM-DFDF yielded an MAE of 3.09 bpm, an ARE of 2.38%, and a VHR of 72.83%. Thus, FSM-SGPS yielded a lower MAE and higher VHR compared with both DFDF and FSM-DFDF. The FSM-SGPS results were statistically different from the results of both DFDF and FSM-DFDF (*p* < 0.001).

**Table 5:** Performance comparison of the DFDF, FSM-DFDF, and FSM-SGPS HR estimation methods for the 24 subjects of the BAMI dataset.

Subject	DFDF		FSM-DFDF			FSM-SGPS		
	MAE (bpm)	ARE (%)	MAE (bpm)	ARE (%)	VHR (%)	MAE (bpm)	ARE (%)	VHR (%)
**1**	11.86	10.41	1.18	0.92	76.60	1.35	1.10	98.08
**2**	9.00	6.31	1.60	1.35	71.47	1.65	1.33	96.79
**3**	4.86	4.18	1.35	1.22	79.38	1.57	1.42	94.00
**4**	17.31	12.86	15.75	11.06	58.65	1.15	0.94	92.63
**5**	25.49	21.99	3.70	3.07	45.83	2.67	2.20	88.46
**6**	**33.06**	**22.37**	**33.88**	**23.00**	**48.68**	**12.35**	**8.51**	**77.94**
**7**	3.90	2.90	1.75	1.39	91.13	1.83	1.43	91.13
**8**	4.81	4.12	1.91	1.66	82.73	2.09	1.81	94.00
**9**	21.30	16.53	1.40	1.07	44.23	1.75	1.34	83.65
**10**	10.56	7.01	1.65	1.10	72.12	1.76	1.17	94.23
**11**	31.28	20.46	1.89	1.45	43.65	1.84	1.31	90.41
**12**	6.58	5.28	1.56	1.45	79.62	1.77	1.64	90.41
**13**	3.15	3.17	1.67	1.64	87.05	1.66	1.61	91.37
**14**	3.62	3.62	1.66	1.76	80.58	1.75	1.84	84.17
**15**	3.30	3.03	3.08	2.75	91.61	3.08	2.75	91.61
**16**	14.71	11.48	2.27	1.87	51.08	2.42	1.94	71.70
**17**	8.59	5.98	1.34	1.00	72.44	1.38	1.00	91.67
**18**	15.55	9.99	1.51	1.11	65.71	1.59	1.13	86.86
**19**	3.95	2.93	1.28	1.03	81.77	1.42	1.12	97.60
**20**	3.17	2.30	1.48	1.13	93.29	1.46	1.11	96.40
**21**	4.17	2.85	1.71	1.25	93.59	1.74	1.26	95.19
**22**	2.42	2.23	1.47	1.34	91.37	1.66	1.52	94.00
**23**	12.54	11.39	1.61	1.41	69.78	1.73	1.54	88.25
**24**	6.17	6.42	1.41	1.53	75.32	1.48	1.59	91.03
**Average**	**10.89**	**8.32**	**3.08**	**2.38**	**72.82**	**2.14**	**1.73**	**90.48**

**Fig 9:**
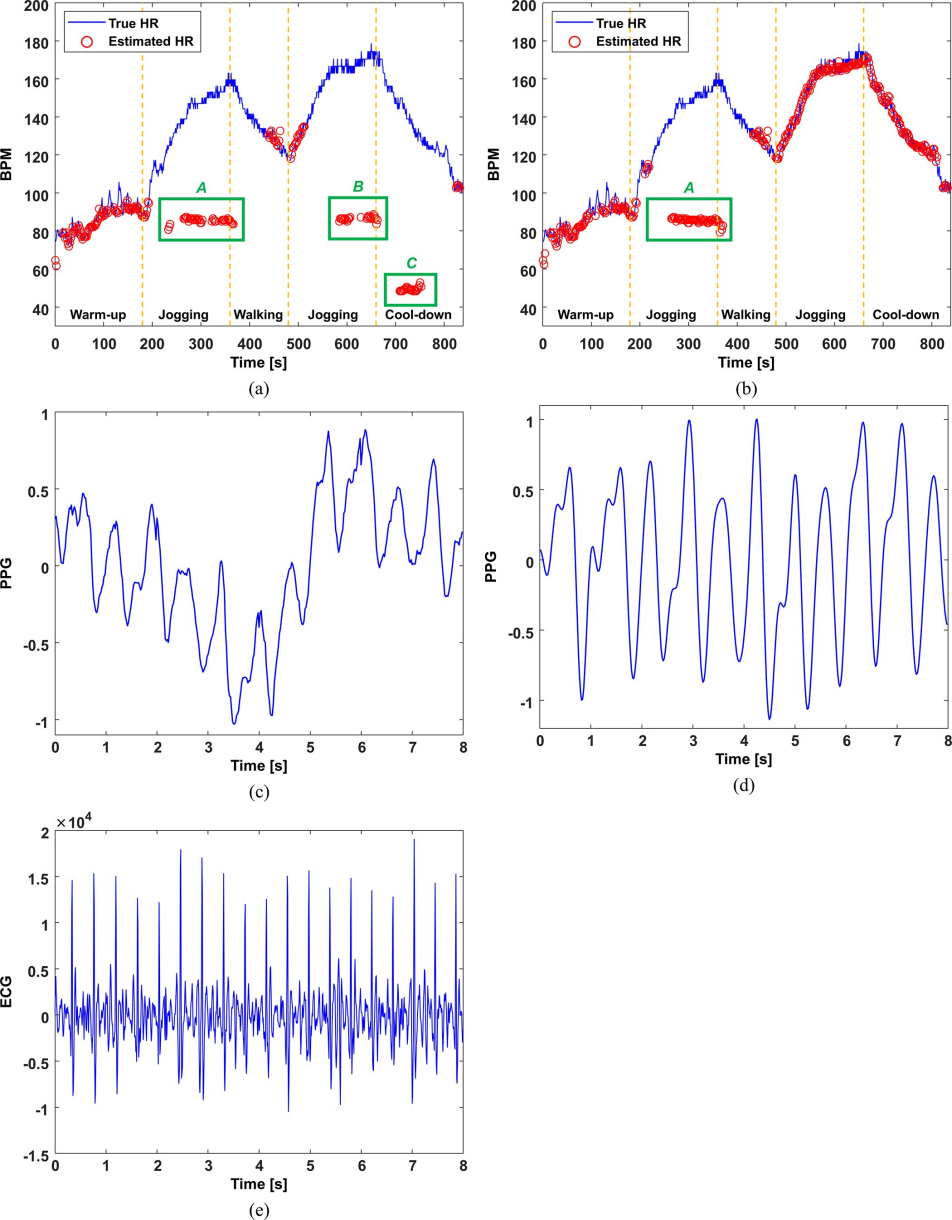
Comparison of the estimated HR trace obtained. (a) FSM-DFDF and (b) FSM-SGPS methods for one subject: (c) example of the measured 8-s PPG signal in region *A*, (d) reconstructed 8-s PPG signal after MA removal followed by inverse FFT, and (e) simultaneously measured 8-s ECG signal.

The results of *subject 6*, whose PPG signals were severely corrupted with MAs, are noteworthy. DFDF produced an extremely high MAE of 33.06 bpm, and FSM-DFDF did not produce any significant accuracy enhancement (MAE = 33.88 bpm). Fig 9(A) shows the estimated HR trace for FSM-DFDF. As can be observed from the figure, the estimated HRs in regions *A*, *B*, and *C* deviate drastically from the true HRs, indicating that the FSM framework validated the estimates in these regions because of the absence of large variations in the estimated HR changes for consecutive windows and consistently high CF values over a long period. Conversely, FSM-SGPS increased the overall accuracy through modification of the power spectrum ***P***_*S*_(*i*), as reflected by the MAE and VHR values (see also Fig 9(B)). The results indicate that the estimation results were improved, especially in regions *B* and *C*. The incorrect estimation results in region *A* will be discussed in the next section.

## Discussion and conclusions

In the pre-processing stage, we down-sampled the signals to 25 Hz. Regarding the effects of the sampling rate, it was reported that the HR estimation performance was nearly the same but the computational time was drastically reduced by down-sampling the signals [20]. Moreover, in [2], the HR estimation performances were compared at different sampling frequencies of 25, 125, 250, and 500 Hz. The results showed that the HR estimation results were similarly accurate for the first 12 subjects of the ISPC dataset, i.e., MAEs of 1.02 bpm (25 Hz), 1.06 bpm (125 Hz), 1.10 bpm (250 Hz), and 1.12 bpm (500 Hz). Our results also showed consistent trends. Even with a different sampling rate, the HR estimation performance was nearly the same. The MAE values with a sampling rate of 125 Hz were 1.21 bpm and 2.15 bpm for the ISPC and BAMI datasets, respectively. Note that the MAE values with a sampling rate of 25 Hz were 1.20 bpm and 2.14 bpm for the ISPC and BAMI datasets, respectively. As down-sampling reduces the computational load without accuracy degradation, most existing algorithms including our method down-sampled the signals to around 25 Hz [1–3, 20–25, 27].

**Fig 10:**
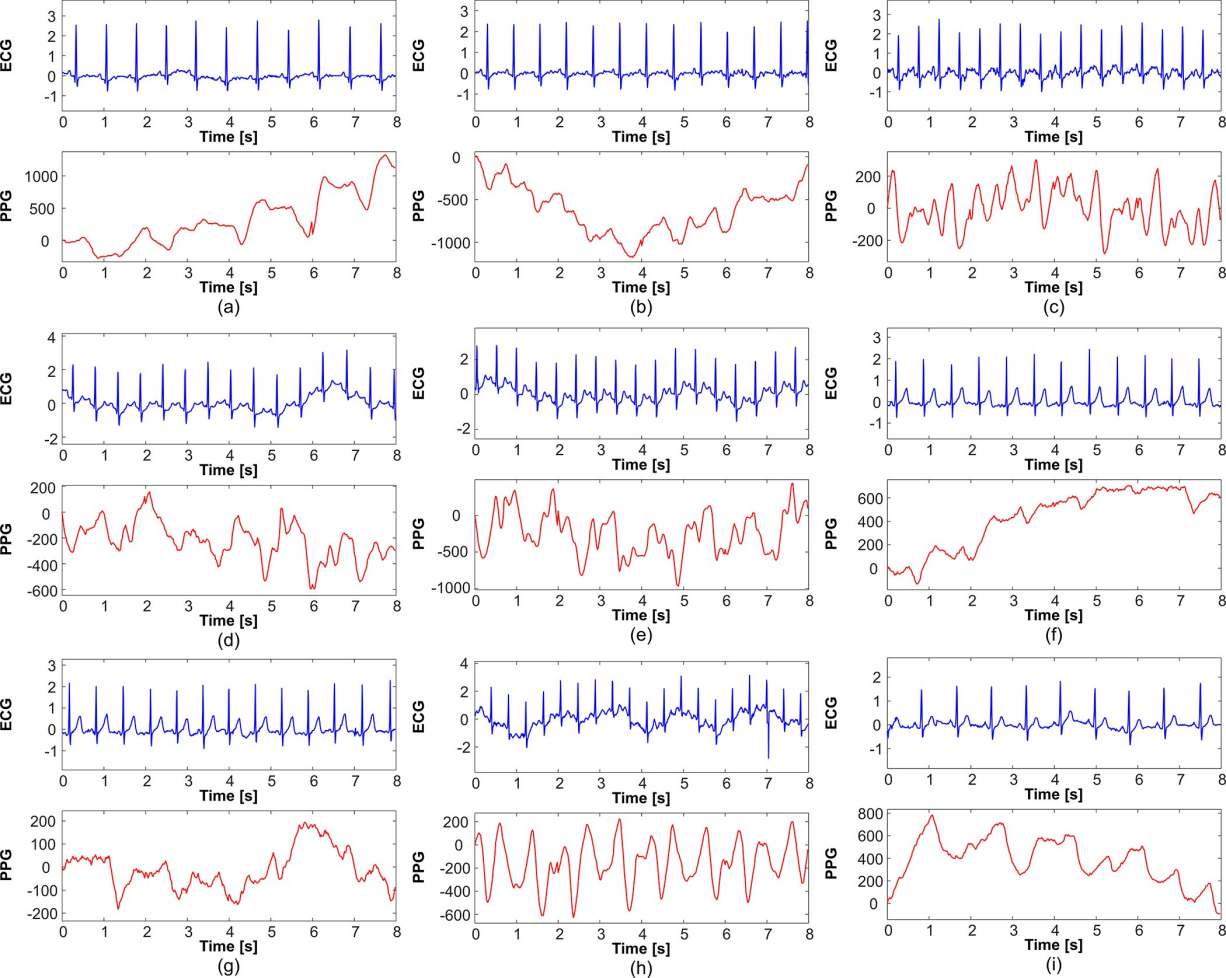
Examples of PPG signals corrupted by MAs. (a)–(i) show PPG signals corrupted by different MAs. There are numerous MA patterns in PPG signals.

There are numerous MA patterns in PPG signals, all of which are unpredictable. Fig 10 shows some examples of corrupted PPG signals along with the simultaneously measured ECG signals during high-intensity exercise. Moreover, in the measured PPG signals, the MAs are shaped similarly to ordinary pulses and are therefore difficult to distinguish. To obtain accurate HR estimation results, considerable research effort has been devoted toward the use of simultaneously measured acceleration signals as noise references in HR estimation algorithms. As numerous MAs are combined with real pulse waves in an unpredictable manner, exact mathematical formulation is difficult. In this study, we simplified the additive relationship between the two spectra. Furthermore, many such approaches have been proposed. For instance, in [25], the MA frequency powers were suppressed by dividing a constant value in the MA frequency range. In [31], the MA frequency power from an acceleration signal was considered as the probability of the event that the corresponding frequency is not the HR while the frequency power from the PPG signal was considered as the probability of the event that the corresponding frequency is the HR. In [21], the frequency peaks in the PPG spectrum were compared with the frequency peaks in the accelerometer spectra, and the HR candidates were eliminated if the peaks were overlapped. We will investigate the relation in future work.

Based on each proposed relationship, the accelerometer-assisted MA cancellation algorithms provided accurate HR estimation results. However, these results were not always accurate. To address this issue, we modified the power spectrum ***P***_*S*_(*i*) by applying a Gaussian kernel function with a mean value of *HR*_*est*_(*i*−1) when the state in the *i*^th^ window was stable. As a result, the MAE values improved to 1.20 bpm from 6.73 bpm for the ISPC dataset and to 2.14 bpm from 10.89 bpm for the BAMI dataset. On the other hand, when the power spectrum modifications were performed for all the windows regardless of the state, the MAEs increased to 1.79 bpm from 1.20 bpm, and to 4.75 bpm from 2.14 bpm, for the ISPC and BAMI datasets, respectively. The AREs also increased to 1.67% from 1.05%, and to 3.55% from 1.73%, for the ISPC and BAMI datasets, respectively. In addition, we found that the VHRs slightly decreased to 77.92% from 78.84%, and to 87.82% from 90.48%, for the ISPC and BAMI datasets, respectively. Thus, the power spectrum modification is more effective when the state in the *i*^th^ window is stable on the basis of the FSM framework.

Compared with FSM-DFDF for the ISPC dataset, FSM-SGPS slightly increased the MAE from 0.99 to 1.20 bpm, whereas it increased the VHR considerably from 57.44% to 78.84%. For the BAMI dataset, the MAE decreased from 3.08 to 2.14 bpm, while the VHR increased from 72.71% to 90.48%. However, as shown in Fig 9(B), the proposed FSM-SGPS algorithm still has some limitations for accurate estimation and validation of all HRs. Its deficiency can be observed when the PPG signal does not reflect the true HR over a long period, even with MA cancellation. The power spectrum ***P***_*S*_(*i*) cannot be modified under this condition owing to the unavailability of the estimation result of the previous window. Fig 9(C) and 9(D) show the 8-s segment example of the measured PPG signal and the corresponding reconstructed PPG signal after MA removal followed by inverse FFT in region *A*, respectively. Fig 9(E) shows the simultaneously measured 8-s ECG signal, in which the R peaks do not correspond to the pulse peaks from the PPG signal. Extremely low SNR causes this condition, which is often encountered when there is a severe pressure change between the photosensor and the measurement site (wrist), with the pressure change completely overwhelming the pure PPG signal. Regardless of how tightly the device is worn on the wrist to avoid pressure changes, this situation may occur under certain circumstances, such as during high-intensity exercise. Hence, there is a need for further investigation of hardware that minimizes pressure changes. Alternatively, a pressure sensor may be embedded in the wearable device and additional MA cancellation can be applied on the basis of its signals.

## References

[1] Zhang Z. Photoplethysmography-based heart rate monitoring in physical activities via joint sparse spectrum reconstruction. IEEE Transactions on Biomedical Engineering. 2015;62(8):1902–10. 10.1109/TBME.2015.2406332 26186747

[2] Khan E, Al Hossain F, Uddin SZ, Alam SK, Hasan MK. A robust heart rate monitoring scheme using photoplethysmographic signals corrupted by intense motion artifacts. IEEE Transactions on Biomedical Engineering. 2016;63(3):550–62. 10.1109/TBME.2015.2466075 26276979

[3] Temko A. Accurate heart rate monitoring during physical exercises using PPG. IEEE Transactions on Biomedical Engineering. 2017;64(9):2016–24. 10.1109/TBME.2017.2676243 28278454

[4] Lee H, Chung H, Ko H, Jeong C, Noh S, Kim C, et al Dedicated cardiac rehabilitation wearable sensor and its clinical potential. PLOS One. 2017;12(10):e0187108 10.1371/journal.pone.0187108 29088260 PMC5663433

[5] Chung H, Ko H, Thap T, Jeong C, Noh S-E, Yoon K-H, et al Smartphone-based cardiac rehabilitation program: Feasibility study. PLOS One. 2016;11(8):e0161268 10.1371/journal.pone.0161268 27551969 PMC4995057

[6] Moser O, Eckstein ML, McCarthy O, Deere R, Bain SC, Haahr HL, et al Heart rate dynamics during cardio-pulmonary exercise testing are associated with glycemic control in individuals with type 1 diabetes. PLOS One. 2018;13(4):e0194750 10.1371/journal.pone.0194750 29608593 PMC5880363

[7] Jouven X, Empana J-P, Schwartz PJ, Desnos M, Courbon D, Ducimetière P. Heart-rate profile during exercise as a predictor of sudden death. New England Journal of Medicine. 2005;352(19):1951–8. 10.1056/NEJMoa043012 15888695

[8] Adabag AS, Grandits GA, Prineas RJ, Crow RS, Bloomfield HE, Neaton JD, et al Relation of heart rate parameters during exercise test to sudden death and all-cause mortality in asymptomatic men. The American Journal of Cardiology. 2008;101(10):1437–43. 10.1016/j.amjcard.2008.01.021 18471455 PMC2440694

[9] Nishime EO, Cole CR, Blackstone EH, Pashkow FJ, Lauer MS. Heart rate recovery and treadmill exercise score as predictors of mortality in patients referred for exercise ECG. JAMA. 2000;284(11):1392–8. 10989401 10.1001/jama.284.11.1392

[10] Jouven X, Schwartz PJ, Escolano S, Straczek C, Tafflet M, Desnos M, et al Excessive heart rate increase during mild mental stress in preparation for exercise predicts sudden death in the general population. European Heart Journal. 2009;30(14):1703–10. 10.1093/eurheartj/ehp160 19401600

[11] Antoun M, Edwards KM, Sweeting J, Ding D. The acute physiological stress response to driving: A systematic review. PLOS One. 2017;12(10):e0185517 10.1371/journal.pone.0185517 29036199 PMC5642886

[12] Merkely B, Roka A. Assessment of heart rate recovery after exercise stress test: implications for cardiac resynchronization therapy. Citeseer. 2011. 10.1093/europace/euq452 21208946

[13] Michael S, Graham KS, Davis GM. Cardiac autonomic responses during exercise and post-exercise recovery using heart rate variability and systolic time intervals—a review. Frontiers in Physiology. 2017;8:301 10.3389/fphys.2017.00301 28611675 PMC5447093

[14] Kim BS, Yoo SK. Motion artifact reduction in photoplethysmography using independent component analysis. IEEE Transactions on Biomedical Engineering. 2006;53(3):566–8. 10.1109/TBME.2005.869784 16532785

[15] Krishnan R, Natarajan B, Warren S. Two-stage approach for detection and reduction of motion artifacts in photoplethysmographic data. IEEE Transactions on Biomedical Engineering. 2010;57(8):1867–76. 10.1109/TBME.2009.2039568 20172800

[16] Madhav KV, Ram MR, Krishna EH, Komalla NR, Reddy KA. Robust extraction of respiratory activity from PPG signals using modified MSPCA. IEEE Transactions on Instrumentation and Measurement. 2013;62(5):1094–106.

[17] Motin MA, Karmakar CK, Palaniswami M. Ensemble empirical mode decomposition with principal component analysis: a novel approach for extracting respiratory rate and heart rate from photoplethysmographic signal. IEEE Journal of Biomedical and Health Informatics. 2018;22(3):766–74. 10.1109/JBHI.2017.2679108 28287994

[18] Elsner JB, Tsonis AA. Singular spectrum analysis: a new tool in time series analysis: Springer Science & Business Media; 2013.

[19] Salehizadeh S, Dao DK, Chong JW, McManus D, Darling C, Mendelson Y, et al Photoplethysmograph signal reconstruction based on a novel motion artifact detection-reduction approach. Part II: Motion and noise artifact removal. Annals of Biomedical Engineering. 2014;42(11):2251–63. 10.1007/s10439-014-1030-8 24823655

[20] Zhang Z, Pi Z, Liu B. TROIKA: A general framework for heart rate monitoring using wrist-type photoplethysmographic signals during intensive physical exercise. IEEE Transactions on Biomedical Engineering. 2015;62(2):522–31. 10.1109/TBME.2014.2359372 25252274

[21] Salehizadeh S, Dao D, Bolkhovsky J, Cho C, Mendelson Y, Chon KH. A novel time-varying spectral filtering algorithm for reconstruction of motion artifact corrupted heart rate signals during intense physical activities using a wearable photoplethysmogram sensor. Sensors. 2015;16(1):10. 10.3390/s16010010 PMC4732043 26703618

[22] Mashhadi MB, Asadi E, Eskandari M, Kiani S, Marvasti F. Heart rate tracking using wrist-type photoplethysmographic (PPG) signals during physical exercise with simultaneous accelerometry. IEEE Signal Processing Letters. 2016;23(2):227–31.

[23] Sun B, Zhang Z. Photoplethysmography-based heart rate monitoring using asymmetric least squares spectrum subtraction and Bayesian decision theory. IEEE Sensors Journal. 2015;15(12):7161–8.

[24] Lee H, Chung H, Ko H, Lee J. Wearable multichannel photoplethysmography framework for heart rate monitoring during intensive exercise. IEEE Sensors Journal. 2018;18(7):2983–93.

[25] Fujita Y, Hiromoto M, Sato T. PARHELIA: Particle filter-based heart rate estimation from photoplethysmographic signals during physical exercise. IEEE Transactions on Biomedical Engineering. 2018;65(1):189–98. 10.1109/TBME.2017.2697911 28459679

[26] Chung H, Lee H, Lee J. Available from: https://github.com/HeewonChung92/GaussianFSM.

[27] Chung H, Lee H, Lee J. Finite state machine framework for instantaneous heart rate validation using wearable photoplethysmography during intensive exercise. IEEE Journal of Biomedical and Health Informatics. 2018. 10.1109/JBHI.2018.2871177 30235152

[28] Zhang Z. Available from: https://sites.google.com/site/researchbyzhang/ieeespcup2015.

[29] Tanaka H, Monahan KD, Seals DR. Age-predicted maximal heart rate revisited. Journal of the American College of Cardiology. 2001;37(1):153–6. 11153730 10.1016/s0735-1097(00)01054-8

[30] Gellish RL, Goslin BR, Olson RE, McDonald A, Russi GD, Moudgil VK. Longitudinal modeling of the relationship between age and maximal heart rate. Medicine and Science in Sports and Exercise. 2007;39(5):822–9. 10.1097/mss.0b013e31803349c6 17468581

[31] Nathan V, Jafari R. Particle filtering and sensor fusion for robust heart rate monitoring using wearable sensors. IEEE Journal of Biomedical and Health Informatics. 2018;22(6):1834–46. 10.1109/JBHI.2017.2783758 29990023

